# Phlorotannin–Alginate Extract from *Nizimuddinia zanardinii* for Melanosis Inhibition and Quality Preservation of Pacific White Shrimp

**DOI:** 10.3390/foods14213736

**Published:** 2025-10-31

**Authors:** Salim Sharifian, Seraj Bita

**Affiliations:** Fisheries Department, Faculty of Marine Sciences, Chabahar Maritime University, Chabahar 99717-78631, Iran; serajbita@yahoo.com

**Keywords:** pacific white shrimp, melanosis, quality preservation, phlorotannins, alginate, ice storage

## Abstract

Phlorotannin–alginate extracts from brown seaweeds offer promising natural solutions for food preservation. This study investigated the extraction, characterization, and application of phlorotannins and alginate from two brown seaweed species, *Sargassum cristaefolium* and *Nizimuddinia zanardinii*, for inhibiting melanosis and preserving quality in Pacific white shrimp during ice storage. Preliminary screening identified *N. zanardinii* methanol extract as superior, yielding the highest phlorotannin content (19.14 ± 0.65 mg Phloroglucinol/g) with potent antioxidant (98.95 ± 0.74% DPPH inhibition) and copper-chelating (73.44 ± 1.64%) activities. Consequently, *N. zanardinii* was selected for subsequent extraction and application studies. Alginate extraction efficiency was 4.73 ± 0.38 g/100 g seaweed, demonstrating moderate antioxidant properties. The extracts effectively inhibited shrimp polyphenol oxidase, with 2% phlorotannins + 1% alginate showing 84.51% inhibition. When applied to shrimp, this combination significantly delayed melanosis development, suppressed microbial growth, and maintained lower pH, total volatile basic nitrogen (TVB-N), and lipid oxidation values during 16 days of ice storage compared to untreated controls. Sensory evaluation confirmed better retention of quality attributes in treated shrimp. These findings demonstrate the potential of *N. zanardinii* phlorotannin–alginate extracts as effective natural preservatives for maintaining shrimp quality during cold storage, offering a sustainable alternative to synthetic additives in seafood processing.

## 1. Introduction

Seafood products, including shrimp, are highly valued and in high demand worldwide. However, their perishable nature makes effective preservation methods crucial to maintaining their quality, safety, and sensory attributes throughout storage and distribution. Shrimp, in particular, are susceptible to various deteriorative processes such as microbial growth, enzymatic reactions, and oxidative deterioration, all of which contribute to quality loss and reduced shelf life. Among the challenges encountered in shrimp preservation, melanosis stands out as a common postharvest problem that significantly affects the visual appeal and market value of the product [1].

Melanosis is characterized by the enzymatic oxidation of phenolic compounds present in the exoskeleton of shrimp, leading to the undesirable darkening of their appearance [2]. This enzymatic browning process not only affects the visual appeal of the shrimp but also serves as a precursor for microbial growth and spoilage, ultimately compromising their overall quality and marketability. Consequently, finding effective strategies to inhibit melanosis and maintain the quality of shrimp during storage is of utmost importance for the seafood industry [3,4].

Several chemical agents, such as sodium metabisulfite, 4-hexylresorcinol, and ascorbic acid, have traditionally been applied to control melanosis in crustaceans [5]. Although these compounds are effective inhibitors of polyphenol oxidase (PPO), their continuous use raises concerns regarding potential toxicity, allergenicity, and adverse effects on consumer health and the environment [1,2,3,4]. Furthermore, chemical treatments may cause undesirable alterations in flavor, texture, or nutritional value and are increasingly restricted due to strict food safety regulations [2].

In recent years, there has been growing interest in the utilization of natural bioactive compounds as alternatives to synthetic additives in food preservation. One such group of compounds is phlorotannins, which are polyphenolic compounds abundant in marine brown algae [4]. Phlorotannins have been recognized for their antioxidant and antimicrobial properties, making them potential candidates for inhibiting enzymatic browning and delaying spoilage in seafood products. Additionally, alginate, a natural polysaccharide extracted from various seaweed species, has been studied for its film-forming ability and potential as a food preservative [5]. Both phlorotannins and alginate have been reported to exhibit inhibitory effects on PPO activity [1,6].

Considering the potential benefits of phlorotannins and alginate, the combination of these compounds in a rich extract form offers a promising solution for inhibiting melanosis and preserving the quality of shrimp during storage. However, specific research investigating the effects of phlorotannin–alginate-rich extract on melanosis inhibition and the overall quality of shrimp during iced storage is limited. The objective of this study was to investigate the effects of a phlorotannin–alginate-rich extract on melanosis development and the quality attributes of Pacific white shrimp (*Litopenaeus vannamei*) during iced storage.

## 2. Materials and Methods

### 2.1. Chemicals and Reagents

All solvents (methanol, ethanol, ethyl acetate, chloroform, dichloromethane, acetonitrile) were of HPLC or analytical grade and purchased from Merck (Darmstadt, Germany). Phloroglucinol (PGH, ≥99%), 2,2-diphenyl-1-picrylhydrazyl (DPPH), Folin–Ciocalteu reagent, L-DOPA (3,4-dihydroxy-L-phenylalanine), copper(II) sulfate, sodium metabisulfite, ascorbic acid, and thiobarbituric acid (TBA) were obtained from Sigma-Aldrich (St. Louis, MO, USA). Plate count agar (PCA) and microbiological media were purchased from Merck. All chemicals were of analytical grade (≥98% purity).

### 2.2. Seaweed Collection

The brown seaweed species *Sargassum cristaefolium* and *Nizimuddinia zanardinii* were manually collected from the intertidal zone of the coastal area near Chabahar city, Iran, in December and November 2024, respectively. Approximately 3 kg of fresh material from each species was collected from three different sites along the coastline to ensure sample representativeness. The collected samples were transported to the laboratory of Chabahar Maritime University for subsequent analysis. Species confirmation was conducted by referencing validated identification keys [7]. Upon arrival at the laboratory, the seaweed samples were first rinsed with freshwater to remove any impurities such as sand, shells, and other debris. Then, the samples were dried in shade to a constant weight. The dried samples were then ground into a fine powder using a household Philips blender (Eindhoven, Netherlands). To prevent degradation of the bioactive compounds present in the seaweed, the powdered samples were stored in airtight zip-lock plastic bags and kept in a freezer at −20 °C until the extraction process.

### 2.3. Preliminary Solvent Screening and Extraction Procedure

To determine the most efficient solvent for the extraction of phlorotannins and alginate from the seaweed samples, 50 g of the powdered samples were combined with 200 mL of the following solvents: 100% methanol, 70% methanol, 30% methanol, 100% water, 100% ethyl acetate, 70% ethyl acetate, and 30% ethyl acetate. The mixtures were shaken at room temperature for two hours using a lab shaker (IKA, Staufen, Germany). Each solution was then filtered through a Whatman filter paper (No. 1) (Maidstone, UK) to remove any insoluble debris. The filtrates were then concentrated using a rotary vacuum evaporator (RV8V-C, IKA, Staufen, Germany) under reduced pressure at 50 °C until a thick paste was obtained. The yield of the extract, the concentration of phlorotannins and DPPH free-radical scavenging activity were determined for each solvent as described by Sharifian et al. [1].

### 2.4. Extraction and Assay of Phlorotannins (PT)

Based on the preliminary screening results showing superior extraction efficiency, 100% methanol and *N. zanardinii* were selected for phlorotannin and alginate extraction. Phlorotannins were extracted, purified, and quantified using a method adapted from Sharifian et al. [1] with minor modifications. Briefly, 500 g of *N. zanardinii* powder was extracted by agitation with 2 L of absolute methanol at ambient temperature for 2 h. The extract was filtered and the solvent removed under reduced pressure to yield the ‘first extract’. The solid residue remaining after filtration, containing residual biomass, was reserved for alginate extraction and stored for subsequent analyses. The first extract was combined with 4 L of chloroform and agitated for approximately 5 min, then phase separation was induced by the addition of 200 mL distilled water. The upper aqueous phase was further partitioned against dichloromethane (DCM) and ethyl acetate. Each fraction was concentrated using a rotary evaporator, producing DCM, ethyl acetate, and aqueous fractions. The residue obtained from the ethyl acetate fraction (designated crude phlorotannins, PT) was kept for further characterization and was also employed as a dipping treatment for shrimp prior to chilled storage. Total phlorotannin content in each extract was determined by the Folin–Ciocalteu assay with phloroglucinol (PGH) as the calibration standard. Absorbance readings were taken at 700 nm using a UV-160A spectrophotometer (Shimadzu, Croissy, France), following Sharifian et al. [1]. The composition of the crude PT was examined by column chromatography and thin-layer chromatography (TLC) on silica gel. Quantification and compositional analysis of the major phlorotannin compounds was performed by HPLC (Knauer, Berlin, Germany) equipped with an Azura photodiode array detector (6.1 L) (Knauer, Berlin, Germany) and an Acclaim™ 120 C18 column (5 μm, 4.6 × 250 mm) (Thermo Fisher Scientific, Waltham, MA, USA). Separation employed a gradient of solvent A (1% formic acid in water) and solvent B (acetonitrile) at a flow rate of 1.0 mL min^−1^ over 60 min with the following gradient program: 0–5 min, 5% B; 5–30 min, 5–40% B (linear); 30–45 min, 40–80% B (linear); 45–50 min, 80% B (isocratic); 50–55 min, 80–5% B (linear); 55–60 min, 5% B (re-equilibration). The injection volume was 15 μL and detection was monitored at 280 nm. The major phlorotannin compounds (eckol, phloroglucinol, dieckol, bieckol, and phlorofucofuroeckol A) were identified based on their characteristic retention times and UV-Vis spectral patterns, which were previously established through column chromatography purification and characterization in our earlier study [1]. Quantification was performed by measuring the relative peak areas at 280 nm, with results expressed as percentage composition of the total phlorotannin content.

### 2.5. Extraction of Alginate

The extraction of alginate from *N. zanardinii* seaweed was performed following the method outlined by Rostami et al. [8]. Fifty grams of seaweed residue obtained from the previous step was combined with 400 mL of sterile distilled water and subjected to agitation on a heat shaker at 65 °C for three hours. This process was repeated twice, and the residues from all three experiments were collected by centrifugation at 5000× *g* for 10 min. To the resulting residue, distilled water was added to bring the volume to one liter. Subsequently, the pH was adjusted to 11 by the addition of 3% Na_2_CO_3_. The resulting mixture was then centrifuged, and the separated liquid portion was mixed with an equal volume of 70% ethanol. After 12 h, the sedimentation that formed was collected as the separated alginate. To remove excess water, the obtained alginate was thoroughly washed with water and ethanol several times. Following the washing steps, the alginate was dried under a hood and collected for further analysis.

### 2.6. Cupric Ion Chelating Activity

Cupric ion chelating activity was assessed following the method described by Wong et al. [9]. The extracts were initially mixed with hexamine-HCl buffer (10 mM, pH 5) at a concentration of 1 mg/mL. One milliliter of the prepared mixture was then combined with 1 milliliter of copper sulfate II solution (0.4 μM) and 100 microliters of TMM (tetramethylmurexide ammonium salt) solution (2 mM). The reaction mixture was incubated at room temperature for 30 min. The absorbance was measured at 460 nm and 530 nm. To determine the chelating capacity, the absorption ratio was calculated and compared against a standard curve prepared using known concentrations of EDTA (0–100 μM) as a positive chelator control. The percentage of copper ion chelation was reported based on the measured chelating activity of the extracts.

### 2.7. Effect of Phlorotannins and Alginate Extract on the Inhibition of Pacific White Shrimp PPO

#### 2.7.1. Shrimp Sample Preparation

Pacific white shrimps (*L. vannamei*) with a size of 55–60 shrimps/kg were obtained from a local supplier in Chabahar, Iran. The shrimps were freshly caught and free from any preservatives. Upon arrival at the laboratory, the shrimps were washed with cold water (1–3 °C) and stored in ice until further use, with a maximum storage time of 2 h.

#### 2.7.2. Extraction of PPO

The extraction of polyphenol oxidase (PPO) was carried out following the method described by Nirmal and Benjakul [10]. Briefly, the cephalothoraxes of twenty whole shrimps were separated, frozen using liquid nitrogen, and then powdered using a Waring commercial blender (Waring, Torrington, CT, USA). The resulting powder was suspended in 150 mL of the extracting buffer (0.05 M sodium phosphate buffer, pH 7.2, containing 1.0 M NaCl and 0.2% Brij-35). After stirring for 30 min at 4 °C, the mixture was centrifuged (30 min, 8000× *g*, 4 °C) with a Rotina 420R refrigerated centrifuge (Tuttlingen, Germany). The obtained supernatant was subjected to ammonium sulfate precipitation to achieve 40% saturation. The precipitated fraction was then collected and dissolved in 0.05 M sodium phosphate buffer, and dialyzed against the same buffer. After removing insoluble materials by centrifugation, the resulting supernatant was collected and used as the “crude PPO extract.”

#### 2.7.3. PPO Inhibitory Activity of Phlorotannins and Alginate

To determine the most effective inhibitor and achieve high inhibition of PPO, various concentrations and combinations of phlorotannins (PT), alginate (AG), and sodium metabisulfite (SM) were evaluated. The tested solutions included: 0.1% PT, 0.5% PT, 1% PT, 1% SM, 1% AG, 1% PT + 0.5% AG, 2% PT + 1% AG, and 1% ascorbic acid (AA). The PPO inhibitory activity of each solution was assessed using a continuous assay with L-DOPA as the substrate, following the method outlined by Nirmal and Benjakul [10]. Briefly, 100 μL of each solution was mixed with the crude PPO extract and incubated for 30 min at room temperature. Subsequently, 400 μL of 0.05 M phosphate buffer (pH 6.0) was added to the mixture, followed by the addition of 600 μL of pre-incubated 15 mM L-DOPA (45 °C). The reaction was conducted at 45 °C, and the rate of dopachrome formation was continuously monitored for 3 min at 475 nm. The enzymatic activity was evaluated based on the rate of increase in absorbance over time. One unit of PPO activity was defined as the amount of enzyme causing a 0.001 rise in absorbance per minute under the assay conditions. The extent of enzyme inhibition was expressed as a percentage, calculated from the equation: Inhibition (%) = [(A − B)/A] × 100, where A is the PPO activity measured in the control sample and B is the activity obtained in the presence of the inhibitor.

### 2.8. Effect of Phlorotannins and Alginate Extract on the Quality of Pacific White Shrimp During Iced Storage

#### 2.8.1. Treatment of Shrimp with Phlorotannins (PT) and Alginate (AG)

Dipping treatments were performed by immersing whole shrimp in solutions containing 1% PT, 1% PT + 0.5% AG, and 2% PT + 1% AG for 10 min at 4 °C. For comparison, a positive control group was prepared by dipping shrimp in a 1.25% sodium metabisulfite (SM) solution at 4 °C, following Nirmal and Benjakul [10]. During all dipping treatments, a shrimp-to-solution ratio of 1:2 (*w*/*v*) was maintained, while untreated shrimp served as the control. After treatment, the shrimp were drained for 5 min and placed in separate polyethylene containers layered with flaked ice. The shrimp-to-ice ratio was kept at 1:2 (*w*/*w*), and the ice was replenished every 12 h. Each container had drainage holes at the base to remove meltwater. At 4-day intervals throughout a 16-day storage period, fifteen shrimp were randomly taken from each treatment group for assessment of melanosis, chemical composition, microbial load, and sensory attributes.

#### 2.8.2. Melanosis Evaluation

Melanosis development was assessed visually by a panel of seven trained evaluators following the procedure of Nirmal and Benjakul [10]. The assessors inspected whole shrimp and assigned scores for melanosis using a 10-point scale, in which 0 corresponded to the absence of visible melanosis and 10 represented severe melanosis. Each shrimp sample was evaluated blindly and independently by the panel. Additionally, the progression of postharvest melanosis was photographically documented throughout the storage period using a Canon IXUS digital camera (Tokyo, Japan) under constant lighting conditions and standardized camera angles. Multiple images were captured for each sample at regular intervals during iced storage to visually complement the panel evaluation data.

#### 2.8.3. pH and Total Volatile Basic Nitrogen (TVB-N) Measurement

Shrimp muscle was homogenized with distilled water at a 1:5 (*w*/*v*) ratio for 1 min and allowed to equilibrate at room temperature for 5 min. The pH of the homogenate was then recorded using a HM-205 pH meter (Tokyo, Japan).

TVB-N was determined according to the method of Kılınç et al. [11] and expressed as mg N per 100 g of shrimp muscle. For this, 10 g of minced shrimp was placed in a distillation flask with 40 mL of distilled water. Magnesium oxide (1 g) and a few drops of silicone antifoam were added before the mixture was distilled. The resulting distillate was collected in a 500 mL conical flask containing 50 mL of 3% boric acid solution with Tashiro indicator (FUJIFILM Wako Pure Chemical Corporation, Osaka, Japan) and titrated with 0.1 N hydrochloric acid. TVB-N content was calculated using the Equation: TVB-N (mg/100 g meat) = ((V × C × 14)/10) × 100; Where V is the titrant volume (mL) and C is the titrant concentration (N).

#### 2.8.4. Peroxide Value (PV) Determination

Lipid extraction was done following the Bligh and Dyer [12] method. 50 g shrimp meat was homogenized with 150 mL chloroform: methanol (2:1 *v*/*v*) using a Waring blender (Waring, Torrington, CT, USA). The homogenate was filtered and the lipid-containing lower phase collected. The solvent was removed using a rotary evaporator and nitrogen flushing.

PV was determined by the AOAC [13] method and expressed in mEq active O_2_/kg lipid. 0.3 g lipid sample was dissolved in 10 mL chloroform-acetic acid and mixed with 1 mL saturated KI solution. After 5 min, 20 mL distilled water was added. The mixture was titrated with 0.1 N sodium thiosulfate until the yellow color disappeared. A volume of 1 mL of 1.5% starch indicator was added and titration continued until the blue color disappeared. A blank with no lipid was run simultaneously.

#### 2.8.5. Thiobarbituric Acid (TBA) Determination

TBA was determined by the method of Benjakul and Bauer [14] and expressed as mg malondialdehyde/kg shrimp meat. 1 g shrimp meat was mixed with 9 mL 0.25 N HCl containing 0.375% TBA and 15% trichloroacetic acid (TCA). The mixture was heated at 100 °C for 10 min then cooled. After centrifugation at 4000× *g* for 20 min, the supernatant absorbance was measured at 532 nm. TBA value was calculated from a malondialdehyde standard curve.

#### 2.8.6. Microbial Analyses

For each treatment, shrimp samples were analyzed to determine total mesophilic (TMC) and psychrotrophic (TPC) bacterial counts. Ten grams of shrimp muscle was homogenized in 90 mL of sterile Butterfield’s phosphate buffer for 3 min using a stomacher. Serial tenfold dilutions were prepared, and bacterial enumeration was carried out on plate count agar (Merck, Darmstadt, Germany). Mesophilic bacteria were incubated at 37 °C for 24–48 h, whereas psychrotrophic bacteria were incubated at 5 °C for 72 h.

#### 2.8.7. Sensory Evaluation

Sensory analysis was performed according to Li et al. [15] using a 7-member trained panel. 25 shrimp from each treatment were evaluated on each sampling day. A 9-point hedonic scale was used where 9 = highest quality and 1 = lowest quality. Scores below 5 were considered unacceptable. Attributes evaluated included appearance, odor, texture and overall acceptability.

### 2.9. Statistical Analysis

All experiments were carried out in triplicate. Data were analyzed by one-way ANOVA using SPSS 13.0 software. Means were compared using Tukey’s test at a significance level of *p* < 0.05. Results were expressed as mean ± standard deviation.

## 3. Results and Discussion

### 3.1. Preliminary Solvent Screening

The extract yield, phlorotannins content, and antioxidant activity of extracts obtained from *S. cristaefolium* and *N. zanardinii* using different solvents are presented in Table 1. For *S. cristaefolium*, the highest extract yield of 5.50 ± 0.29 g/100 g seaweed was obtained using 70% methanol, while 100% methanol resulted in an extract yield of 4.23 ± 0.11 g/100 g seaweed. However, the phlorotannins content and antioxidant activity determined by DPPH assay were higher for the 100% methanol extract (6.39 ± 0.07 mg PHG/g and 52.63 ± 0.99%, respectively) compared to the 70% methanol extract (5.33 ± 0.09 mg PHG/g and 48.08 ± 0.96%, respectively). For *N. zanardinii*, the 100% methanol extract provided the highest extract yield (6.43 ± 0.09 g/100 g seaweed), phlorotannins content (9.39 ± 0.27 mg PHG/g) and DPPH radical scavenging activity (73.27 ± 0.94%). The 100% water extract for both seaweeds showed the lowest phlorotannins content and antioxidant activity. Ethyl acetate extracts demonstrated high phlorotannins content and antioxidant activity; however, the extract yields were low compared to aqueous methanol extracts. Based on the preliminary screening results, 100% methanol was selected as the most suitable solvent for further extraction of phlorotannins and alginate from *N. zanardinii*, as it provided high extract yield and maximum phlorotannins content with potent antioxidant activity. Previous studies have also reported superior extraction of seaweed phlorotannins using aqueous methanol compared to other solvents [16,17]. The high polarity of methanol allows effective extraction of the phenolic components responsible for the observed bioactivities.

### 3.2. Phlorotannins Content and Bioactivities of N. zanardinii Extracts

**Figure 1:**
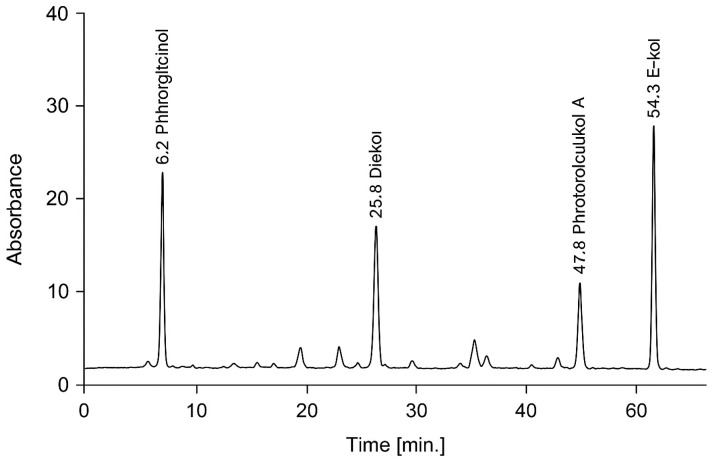
High-performance liquid chromatography profile of phlorotannin compounds isolated from *Nizimuddiana zanardinii.*

The extract yield, phlorotannins content, antioxidant activity (DPPH assay), and copper-chelating activity of the initial methanol extract and subsequent fractions of *N. zanardinii* are presented in Table 2. The first methanol extract showed the highest extract yield of 6.43 ± 0.09 g/100 g seaweed as well as potent antioxidant activity of 73.27 ± 0.94% DPPH inhibition and moderate copper-chelating ability (43.45 ± 1.17% inhibition). Among the fractions, the ethyl acetate fraction demonstrated the maximum phlorotannins content of 19.14 ± 0.65 mg PGH/g extract as well as the highest antioxidant (98.95 ± 0.74% DPPH inhibition) and copper-chelating activities (73.44 ± 1.64% inhibition). The dichloromethane fraction also showed relatively high phlorotannins content and antioxidant activity. In contrast, the hexane fraction contained negligible phlorotannins and poor bioactivities. The high concentration of phlorotannins in the *N. zanardinii* ethyl acetate fraction correlates well with several previous studies that have reported ethyl acetate as an efficient solvent for enriching phlorotannins from brown algae. For example, Li et al. [18] found that sequential extraction with acetone and ethyl acetate yielded an extract from *Sargassum fusiforme* with the highest phlorotannins content (88.48 ± 0.30 mg PGE/100 mg extract) and antioxidant activity. Similarly, Almeida et al. [19] showed that ethyl acetate extraction provided enrichment of low molecular weight phlorotannins from *Fucus spiralis*. The relatively non-polar nature of ethyl acetate allows preferential solubilization of low- and medium-molecular-weight phlorotannins, leaving higher molecular weight compounds concentrated in the residue [20]. Ethyl acetate fractions from *Fucus vesiculosus* [21], *Sargassum muticum* [22] and *Scytosiphon lomentaria* [23] have also shown high phlorotannin contents up to 24% dry weight. The composition of the phlorotannins extracted from *N. zanardinii* was further analyzed by HPLC, as shown in Figure 1. The chromatogram indicates that the extract contained a diversity of phlorotannin compounds. Quantification revealed that the major components were eckol (19.2%), phloroglucinol (16.3%), dieckol (9.9%), bieckol (8.6%), and phlorofucofuroeckol A (8.4%), along with 37.6% of other minor compounds. This profile is consistent with previous characterization of phlorotannins from brown algae, where eckol, phloroglucinol, fuhalols and other phlorotannin derivatives are commonly found [1,24]. The variety of mono-, di-, and trimer phlorotannin structures likely contributes to the potent antioxidant and bioactive properties observed for the *N. zanardinii* extracts.

### 3.3. Yield and Bioactivities of Alginate from N. zanardinii

In addition to phlorotannins, the polysaccharide alginate was extracted from *N. zanardinii* and its yield and antioxidant properties were characterized. An alginate extraction efficiency of 4.73 ± 0.38 g per 100 g of seaweed was achieved. The purified alginate extract contained appreciable levels of phenolic compounds (6.64 ± 0.41 mg PGH/g). This phenolic content correlated with moderate antioxidant activities, as the alginate demonstrated 23.07 ± 1.67% DPPH radical scavenging and 43.47 ± 0.99% copper-chelating abilities. The obtaining of alginate with inherent antioxidant capacity has been reported previously in brown algae. Borazjani et al. [25] extracted alginate from *Sargassum* spp. that showed DPPH radical scavenging in the range of 39–66%. Antioxidant alginates isolated from *Lessonia trabeculata* [26] and *Sargassum wightii* [27] also exhibited phenolic contents of 4–8 mg Gallic acid equivalent/g. The phenolic compounds are likely integrated into the alginate polysaccharide matrix through covalent linkages [28], conferring its natural bioactive properties.

### 3.4. PPO Inhibitory Activity of Phlorotannins and Alginate

The inhibitory effects of phlorotannins, alginate, and their combination on polyphenol oxidase (PPO) from Pacific white shrimp are summarized in Table 3. At 0.1%, the phlorotannin extract exhibited weak PPO inhibition of 13.30%. However, increasing the concentration to 0.5% and 1% significantly enhanced the PPO inhibitory activity to 32.67% and 68.37%, respectively. The inhibitory effect of phlorotannins was concentration-dependent. Similarly, the synergistic blend of 1% phlorotannins + 0.5% alginate showed 76.65% PPO inhibition, surpassing their individual activities. Of the treatments tested, 2% phlorotannins + 1% alginate exhibited the highest PPO inhibition at 84.51%, which was on par with 1% ascorbic acid (72.43%). This indicates the synergistic combination of phlorotannins and alginate can match or even exceed the PPO inhibitory effect of the common reference standard ascorbic acid. The inhibitory effects of phlorotannins, alginate, and their combinations on polyphenol oxidase (PPO) have been documented in several studies. Kurihara and Kujira [29] reported concentration-dependent PPO inhibition reaching 68–85% for phlorotannin extracts from the brown algae *Colpomenia bullosa* at levels of 0.5–1 mg/mL. Li et al. [30] found a synergistic effect of polyphenols and polysaccharides in inhibiting PPO from the Pacific white shrimp. Phlorotannins are thought to inhibit PPO activity through several mechanisms, including chelation of the copper cofactor needed for PPO function, direct binding to enzymes to block active sites, and interaction with PPO substrates such as phenols to render them unavailable [1]. The diverse structures of phlorotannins allow for multiple modes of action. Alginate has also exhibited PPO inhibition. Xu et al. [31] found that the use of 2% sodium alginate during refrigerated storage could inhibit PPO activity. Combining alginate with organic acids has been found to further improve PPO inhibition compared to individual compounds [32]. Alginate’s polysaccharide structure may enable interactions with PPO proteins. Furthermore, when combined with polyphenolic compounds, alginate could confer additional antioxidant effects against PPO. However, further research is needed to fully characterize alginate’s specific mechanisms of PPO inhibition and how these may be influenced when coupled with different phenolic components.

### 3.5. Effect of Phlorotannins and Alginate Extract on the Quality of Pacific White Shrimp During Iced Storage

#### 3.5.1. Melanosis Changes

**Figure 2:**
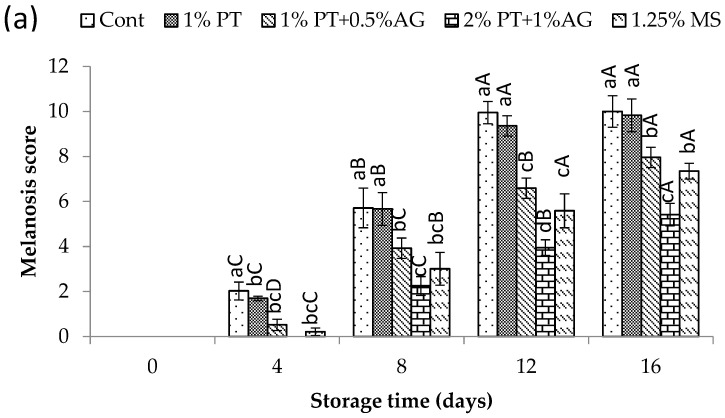

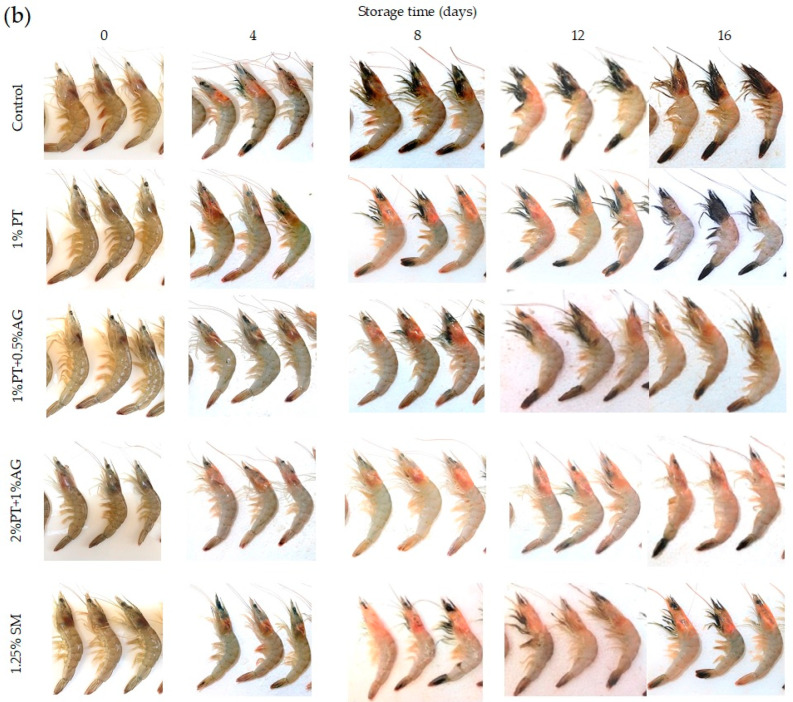
(**a**) Melanosis score and (**b**) visual progression in Pacific white shrimp subjected to phlorotannins (PT) and alginate (AG) treatments throughout 16 days of iced storage. Different uppercase letters on the bars indicate significant differences (*p* < 0.05) as a function of storage time. Different lowercase letters on the bars indicate significant differences (*p* < 0.05) as a function of treatments.

Figure 2a,b present the effect of phlorotannins and alginate treatment on inhibiting melanosis formation in Pacific white shrimp during 16 days of iced storage. Over the storage period, the visual melanosis score increased for all treatments as seen in Figure 2a. However, shrimp treated with 2% phlorotannins (PT) + 1% alginate (AG) exhibited significantly lower scores compared to the control (*p* < 0.05). After 16 days, the melanosis score reached 6.93 in the control versus only 2.67 with 2% PT + 1% AG (*p* < 0.05). The lower melanosis scores align with our previous study, where we found phlorotannin extracts decreased melanosis in chilled shrimp [1]. Similarly, Shiekh et al. [3] demonstrated that immersion in polyphenol-enriched Chamuang leaf extract (1% *w*/*w*) effectively retarded melanosis development in Pacific white shrimp relative to untreated samples. Figure 2b shows representative images of melanosis progression. The untreated control displayed considerable black spot formation by day 16. In contrast, shrimp treated with 2% PT + 1% AG showed minimal darkening, comparable to 1.25% SM.

#### 3.5.2. Physicochemical Changes

**Figure 3:**
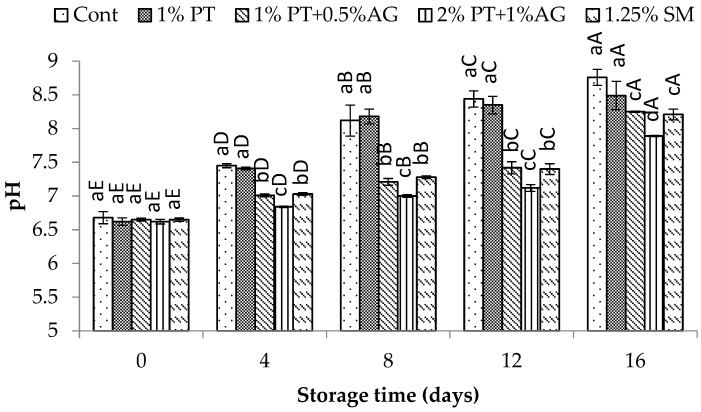
pH variations in Pacific white shrimp subjected to phlorotannins (PT) and alginate (AG) treatments throughout 16 days of iced storage. Key: refer to Figure 2 caption.

**Figure 4:**
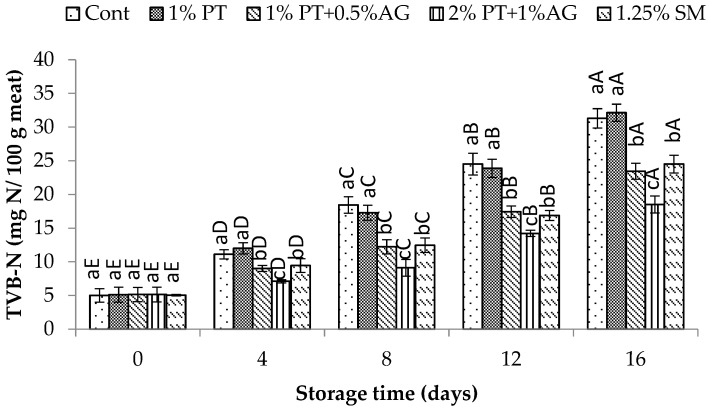
TVB-N variations in Pacific white shrimp subjected to phlorotannins (PT) and alginate (AG) treatments throughout 16 days of iced storage. Key: refer to Figure 2 caption.

**Figure 5:**
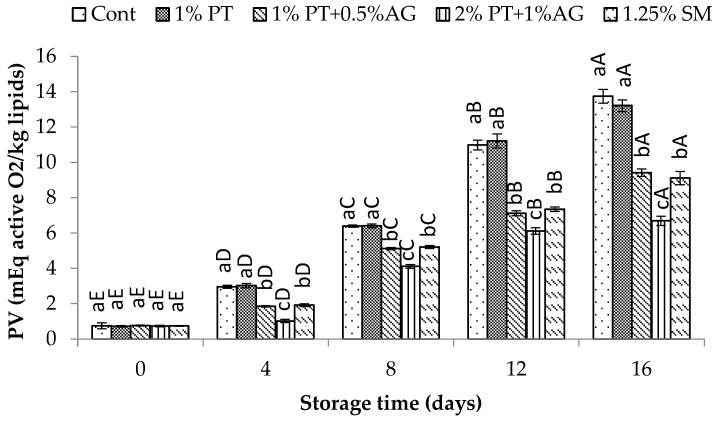
PV variations in Pacific white shrimp subjected to phlorotannins (PT) and alginate (AG) treatments throughout 16 days of iced storage. Key: refer to Figure 2 caption.

**Figure 6:**
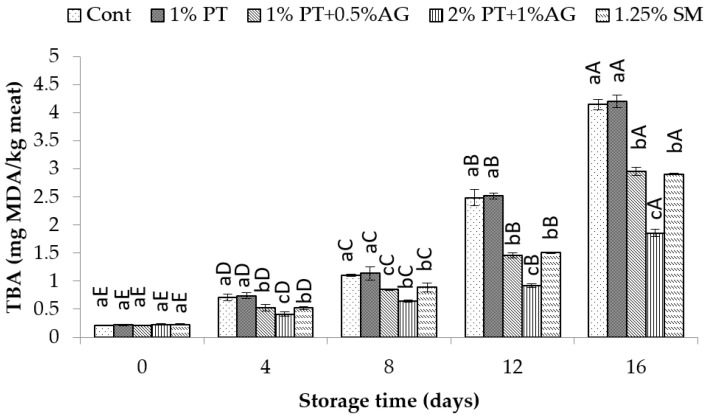
TBA variations in Pacific white shrimp subjected to phlorotannins (PT) and alginate (AG) treatments throughout 16 days of iced storage. Key: refer to Figure 2 caption.

**Figure 7:**
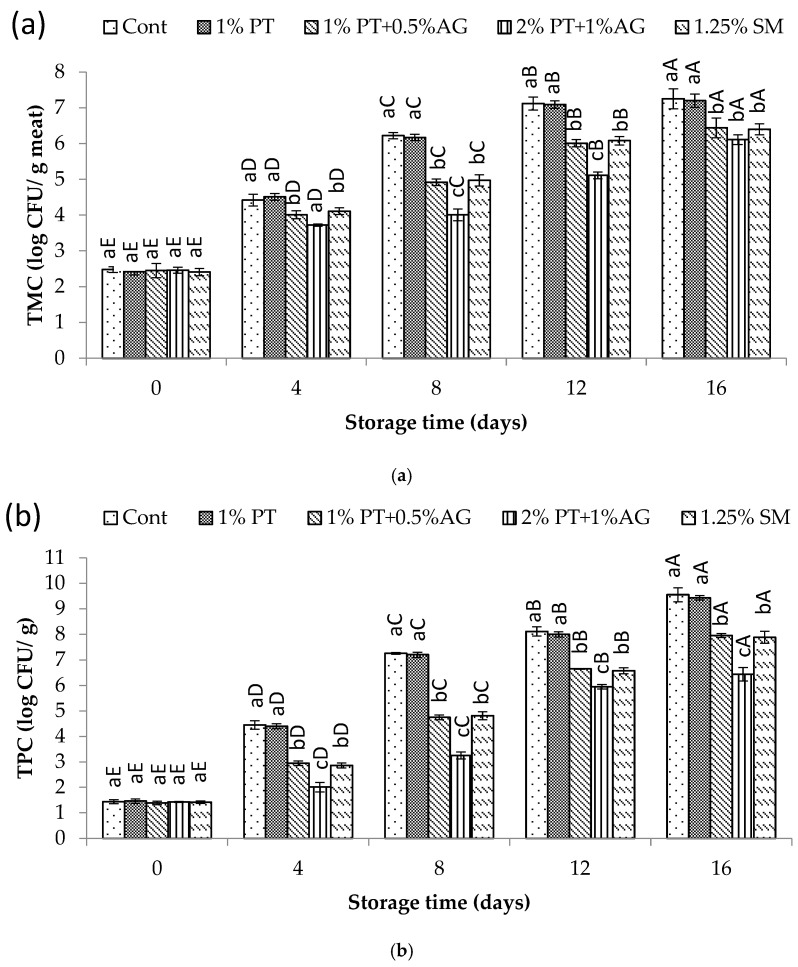
(**a**) Mesophilic (TMC) and (**b**) psychrophilic bacterial count (TPC) variations in Pacific white shrimp subjected to phlorotannins (PT) and alginate (AG) treatments throughout 16 days of iced storage. Key: refer to Figure 2 caption.

The effects of phlorotannins and alginate treatment on quality indices including pH, TVB-N, PV, and TBA in shrimp during chilled storage are presented in Figure 3, Figure 4, Figure 5 and Figure 6. The pH of shrimp muscle gradually increased over the 16-day storage period (Figure 3). However, shrimp dipped in 2% PT + 1% AG maintained a significantly lower pH from day 8 onwards compared to control (*p* < 0.05). By day 16, the pH had reached 7.8 ± 0.11 in control versus 7.3 ± 0.09 in treated shrimp (*p* < 0.05). The gradual pH increase in shrimp muscle during chilled storage indicates accumulation of alkaline compounds like ammonia from protein breakdown. The slower pH rise in PT + AG-treated shrimp aligned with their lower TVB-N values (Figure 4) and microbial counts (Figure 7a,b), indicating inhibited protein decomposition and bacterial growth. Other studies on polyphenolic extracts in shrimp also found effective pH control. Li et al. [30] reported smaller increases in pH for Pacific white shrimp treated with 5 g/L polyphenol and 8 g/L polysaccharides extracted from *Porphyra yezoensis* (red algae) compared to controls during refrigerated storage. They attributed the lower pH to antimicrobial effects of phenolic compounds. Similarly, the phlorotannins and alginate in the current study likely suppressed microbial activity, slowing the pH increase.

The progression of TVB-N in shrimp samples throughout the storage is exhibited in Figure 4. A general increase in TVB-N values was observed over time for all treatments, indicative of on-going protein degradation. However, shrimp treated with PT + AG maintained significantly lower (*p* < 0.05) TVB-N levels compared to the control from Day 8 onwards. By the end of storage on Day 16, TVB-N had reached 46.8 ± 2.3 mg N/100 g in the control shrimp, while 2% PT + 1% AG-treated shrimp showed only 28.4 ± 1.9 mg N/100 g (*p* < 0.05). Previous research has demonstrated that phlorotannins exhibit antimicrobial properties against diverse seafood spoilage bacteria [33]. In this study, the inhibitory effect of PT + AG treatment on TVB-N accumulation likely resulted from the antimicrobial capacity of phlorotannins to inhibit microbial proliferation and associated proteolytic degradation leading to TVB-N formation, as supported by reported reductions in total viable count and psychrotrophic bacteria in seafood imparted with phlorotannin extracts [1]. Additionally, alginate components may have formed an antioxidant film on shrimp surfaces in combination with phlorotannins, acting as a barrier against microbial infiltration and enzymatic deterioration of proteins over time [30]. Hence, through multifactorial mechanisms, the 2% PT + 1% AG treatment very effectively controlled TVB-N accumulation and maintained better protein quality in Pacific white shrimp compared to untreated samples.

As seen in Figure 5, PV increased gradually during storage for all samples due to lipid oxidation. However, 2% PT + 1% AG-treated shrimp maintained significantly lower PV compared to control (*p* < 0.05). By day 16, PV was 20.5 ± 1.2 mEq active O^2^/kg lipid in control versus 12.3 ± 0.8 mEq active O_2_/kg lipid in treated shrimp. Similarly, TBA values increased over time for all samples as a result of secondary lipid oxidation products (Figure 6). However, PT + AG-treated shrimp exhibited significantly lower TBA content, indicating reduced lipid oxidation. By day 16, TBA had reached 5.8 ± 0.4 mg MDA/kg in control compared to 3.2 ± 0.2 mg MDA/kg in treated shrimp (*p* < 0.05). The reduced PV and TBA values observed in 2% PT + 1% AG treatment likely resulted from the synergistic antioxidant and antimicrobial properties of phlorotannins and alginate. Previous studies have reported inhibitory effects of phlorotannins on lipid oxidation in shrimp and fish [1]. Additionally, alginate films enriched with phlorotannins exhibited improved antioxidant activity in foods [34]. Phlorotannins and alginate likely acted synergistically to suppress auto-oxidation and microbial proliferation, maintaining lower levels of protein deterioration and lipid oxidation in shrimp over the 16-day iced storage period.

#### 3.5.3. Microbial Changes

The changes in total mesophilic bacteria (TMC) and psychrotrophic bacteria counts (TPC) during storage are shown in Figure 7a,b. In general, bacterial numbers increased with storage time for all samples. However, shrimp treated with 2% PT + 1% AG demonstrated significantly lower (*p* < 0.05) microbial growth compared to the untreated control. As seen in Figure 7a, TMC in the control shrimp exceeded 7 log CFU/g on Day 16, whereas 2% PT + 1% AG-treated shrimp showed only 5.8 log CFU/g at the same time. Similarly, for TPC, Figure 7b illustrates counts reaching over 8 log CFU/g in the control versus 6.3 log CFU/g in treated shrimp after 16 days of storage. The observed reduction in bacterial growth with 2% PT + 1% AG treatment can be attributed to the antimicrobial action of phlorotannins and alginate. Previous studies have reported that phlorotannin-rich extracts are effective against both Gram-positive and Gram-negative seafood spoilage bacteria [35]. This antibiotic property likely helped suppress the proliferation of TMC and TPC in shrimp muscle throughout iced storage. Additionally, the alginate component may have formed a protective coating on treated shrimp, presenting a physical barrier against microbial attachment and entry [30]. Overall, through synergistic antimicrobial effects, the combination of phlorotannins and alginate very effectively controlled the outgrowth of mesophilic and psychrotrophic bacteria, thereby maintaining better microbial quality of Pacific white shrimp compared to the untreated control during 16 days of chilled storage.

#### 3.5.4. Sensory Evaluation

**Figure 8:**
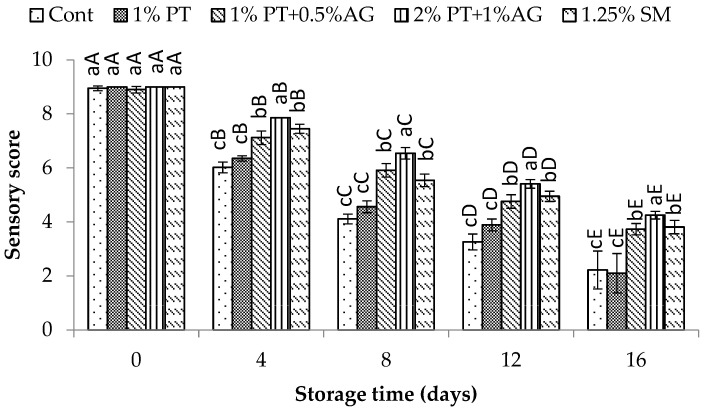
Sensory evaluation scores of Pacific white shrimp subjected to phlorotannins (PT) and alginate (AG) treatments throughout 16 days of iced storage. Key: refer to Figure 2 caption.

Changes in sensory scores of Pacific white shrimp treated without and with 2% PT + 1% AG during 16 days of iced storage are shown in Figure 8. Overall, the sensory scores of all treatments declined throughout storage, with the fastest rate of decline observed in the untreated control shrimp. Initially at Day 0, there were no significant differences (*p* > 0.05) between treatments and scores were within the “high quality” range for all shrimp. After 8 days of storage, scores of the control dropped to 4.71, reaching the unacceptable level below 5. Shrimp treated with 2% PT + 1% AG maintained acceptable scores above 5 until Day 12. On Day 12, untreated shrimp exhibited noticeable quality deterioration through unpleasant odor and soft texture. Meanwhile, treated shrimp still appeared fresh with acceptable aroma and firmness. Shrimp subjected to 2% PT + 1% AG treatment demonstrated superior sensory ratings, retaining acceptable quality through Day 12. These sensory results coincided well with slower melanosis development, lower microbial growth and reduced biochemical changes observed in 2% PT + 1% AG-treated shrimp. Therefore, 2% PT + 1% AG treatment extended the shelf-life of Pacific white shrimp by 4 additional days compared to the control, likely attributable to antioxidant and antimicrobial effects prolonging freshness.

The shelf-life extension and preservation efficacy achieved in the present study compare favorably with other natural plant extract-based methods reported in the recent literature for Pacific white shrimp preservation. Ahmad et al. [36] evaluated ethanolic Thai indigenous leaf extracts and found that 1% galangal leaf extract extended the refrigerated shelf-life of Pacific white shrimp by 3–4 days compared to untreated controls, which is consistent with the 4-day extension observed with 2% PT + 1% AG treatment in the current study. Their study also reported effective melanosis inhibition and maintenance of sensory quality attributes similar to our findings. Likewise, Shiekh et al. [3] demonstrated that 1% Chamuang (*Garcinia cowa* Roxb.) leaf extract successfully retarded melanosis development and preserved sensory quality of Pacific white shrimp during refrigerated storage for up to 12 days, comparable to the results obtained with phlorotannin–alginate treatment. The similar preservation performance of brown seaweed-derived phlorotannin–alginate extracts compared to these terrestrial plant extracts highlights the potential of marine-sourced bioactive compounds as effective and sustainable alternatives for seafood preservation. Furthermore, the synergistic combination of phlorotannins with alginate may offer additional advantages through enhanced film-forming properties and multifunctional bioactivities including antioxidant, antimicrobial, and metal chelating effects, making seaweed extracts particularly promising for commercial seafood processing applications.

## 4. Conclusions

The results of this study demonstrated the potential of a phlorotannin–alginate-rich extract from the brown seaweed *Nizimuddinia zanardinii* to inhibit melanosis and preserve the quality of Pacific white shrimp during ice storage. Following preliminary screening of two brown seaweed species (*Sargassum cristaefolium* and *N. zanardinii*) with different solvent systems, *N. zanardinii* extracted with 100% methanol was selected due to its superior phlorotannin content and bioactivities. The extract exhibited a high concentration of phlorotannins as well as potent antioxidant and metal chelating activities. It was found to effectively inhibit the enzymatic activity of polyphenol oxidase (PPO) from shrimp in a concentration-dependent manner. A synergistic combination of 2% phlorotannins and 1% alginate provided over 84% PPO inhibition, comparable to the standard preservative ascorbic acid. When applied as a postharvest treatment for shrimp, the phlorotannin–alginate blend was successful in delaying melanosis development and maintained lower melanosis scores throughout 16 days of chilled storage compared to the untreated control. It also retarded the microbial, chemical and lipid oxidation spoilage of shrimp, as evidenced through suppressed pH, TVB-N, TBA and peroxide values. Sensory analysis further confirmed the better retention of visual appeal, odor and texture attributes among treated shrimp. In conclusion, the phlorotannin–alginate-rich extract from *N. zanardinii* demonstrated great promise as an effective natural alternative for controlling melanosis and enhancing the shelf-life of Pacific white shrimp during ice storage. With further optimization, this approach can potentially be incorporated into industrial processing for sustainable shrimp preservation under iced storage conditions.

## Figures and Tables

**Table 1:** Extract yield, phlorotannin (PT) content and antioxidant activity of *Sargassum cristaefolium* and *Nizimudiana zanardinii* extracts using different solvents.

	*Sargassum cristaefolium*			*Nizimudiana zanardinii*		
Extraction Solvent	Extract Yield ^1^	PT Content ^2^	DPPH Assay ^3^	Extract Yield	PT Content	DPPH Assay
MeOH100%	4.23 ± 0.11 ^b^*	6.39 ± 0.07 ^c^	52.63 ± 0.99 ^b^	6.43 ± 0.09 ^a^	9.39 ± 0.27 ^b^	73.27 ± 0.94 ^b^
MeOH70%	5.50 ± 0.29 ^a^	5.33 ± 0.09 ^d^	48.08 ± 0.96 ^c^	5.05 ± 0.09 ^b^	6.40 ± 0.14 ^d^	52.48 ± 0.81 ^d^
MeOH30%	3.73 ± 0.11 ^c^	2.23 ± 0.11 ^e^	25.35 ± 0.70 ^d^	4.41 ± 0.21 ^c^	4.56 ± 0.11 ^e^	45.56 ± 0.93 ^e^
H2O100%	3.74 ± 0.08 ^c^	1.41 ± 0.15 ^f^	15.55 ± 0.61 ^f^	4.51 ± 0.22 ^c^	2.59 ± 0.17 ^g^	22.56 ± 0.95 ^g^
EtOAc100%	2.19 ± 0.11 ^f^	8.56 ± 0.08 ^a^	60.63 ± 0.54 ^a^	3.57 ± 0.14 ^d^	12.46 ± 0.14 ^a^	84.38 ± 1.14 ^a^
EtOAc70%	2.78 ± 0.05 ^e^	6.43 ± 0.10 ^b^	53.90 ± 0.65 ^b^	2.35 ± 0.09 ^e^	8.36 ± 0.17 ^c^	66.92 ± 0.63 ^c^
EtOAc30%	3.26 ± 0.06 ^d^	2.37 ± 0.10 ^e^	18.65 ± 0.72 ^e^	2.88 ± 0.07 ^e^	3.62 ± 0.09 ^f^	27.74 ± 0.95 ^f^

^1^ Extract yield: g extract/100 g seaweed; ^2^ Phlorotannins content: mg phloroglucinol/g extract; ^3^ DPPH radical scavenging: % inhibition; All data reported as mean ± SD. * Different lowercase letters in the same column indicate significant differences (*p* < 0.05).

**Table 2:** Extract yield, phlorotannins (PT) content, DPPH and copper-chelating activity of *Nizimudiana zanardinii* extracts.

Fractions	Extract Yield ^1^	PT Content ^2^	DPPH Assay ^3^	Copper Chelating ^3^
First extract	6.43 ± 0.09 ^a^*	9.39 ± 0.27 ^b^	73.27 ± 0.94 ^b^	43.45 ± 1.17 ^b^
TCM fraction	0.28 ± 0.03 ^e^	1.35 ± 0.96 ^d^	5.98 ± 0.43 ^e^	2.34 ± 1.05 ^e^
DCM faction	0.85 ± 0.04 ^d^	5.05 ± 1.03 ^c^	52.36 ± 0.81 ^c^	34.42 ± 1.55 ^c^
EtOAc fraction	2.54 ± 0.12 ^b^	19.14 ± 0.65 ^a^	98.95 ± 0.74 ^a^	73.44 ± 1.64 ^a^
Aqueous fraction	2.16 ± 0.15 ^c^	1.51 ± 0.53 ^d^	13.66 ± 1.06 ^d^	11.43 ± 0.88 ^d^

^1^ Extract yield: g extract/100 g seaweed; ^2^ Phlorotannins content: mg phloroglucinol/g extract; ^3^ DPPH radical scavenging and copper-chelating activity: % inhibition. All antioxidant assays were conducted at a fixed extract concentration of 1 mg/mL. All data reported as mean ± SD. * Different lowercase letters in the same column indicate significant differences (*p* < 0.05).

**Table 3:** PPO inhibitory activity of phlorotannins and alginate extracted from *Nizimudiana zanardinii.*

	0.1% PT	0.5% PT	1% PT	1% SM	1% AG	1% PT + 0.5% AG	2% PT + 1% AG	1% AA
PPO inhibition	13.30 ± 0.85 ^g^	32.67 ± 1.22 ^e^	68.37 ± 0.65 ^d^	67.65 ± 1.42 ^d^	27.39 ± 1.05 ^f^	76.65 ± 1.27 ^b^	84.51 ± 2.15 ^a^	72.43 ± 1.42 ^c^

PT: phlorotannins; SM: sodium metabisulfite; AG: alginate; AA: ascorbic acid. All data reported as mean ± SD. Different lowercase letters indicate significant differences (*p* < 0.05).

## Data Availability

The original contributions presented in this study are included in the article. Further inquiries can be directed to the corresponding author.
